# Reversible pulmonary artery hypertension in association with interferon-beta therapy for multiple sclerosis

**Published:** 2016-01-05

**Authors:** Somayyeh Baghizadeh, Mohammad Ali Sahraian, Mojgan Ghahari

**Affiliations:** 1 Iranian Center of Neurological Research, Iranian Headache Association, Sina Hospital, Tehran University of Medical Sciences ,Tehran, Iran; 2 Department of Neurology, School of Medicine AND Sina Hospital, Tehran University of Medical Sciences, Tehran, Iran; 3 Iran Multiple Sclerosis Society, Tehran, Iran

**Keywords:** Multiple Sclerosis, Pulmonary Arterial Hypertension, Beta-Interferon

The patient was a 32-year-old woman referred to a neurologist in 2008 for walking difficulty and easy fatigue. Physical examination revealed spastic paraparesis. The patient had history of some neurological symptoms since 2003 which were neglected because of little functional impairment and self-limitation. Brain and cervical magnetic resonance imaging (MRI) fulfilled Barkhof criteria for multiple sclerosis (MS). A thorough paraclinical investigation was done and was negative for any other possible explanation for her clinical and MRI presentation. Treatment with subcutaneous interferon beta 1a (IFN-β1a, REBIF@) three times a week began with the diagnosis of relapsing-remitting MS (RRMS). She developed weakness of lower extremities after two months that partially responded to intravenous (IV) corticosteroids. 

She was referred to our center due to increased disability score during eight months period despite regular subcutaneous IFN-β1a injections without any obvious relapse. She also suffered from tachycardia, palpitation and easy fatigue. The treatment regimen changed to mitoxantrone with the impression of secondary progressive course, also propranolol started for palpitation. Baseline echocardiography before mitoxantrone injection showed an ejection fraction of 62% for left ventricle, with moderate tricuspid regurgitation and moderate pulmonary artery hypertension (PAH) with pulmonary artery pressure (PA pressure) of 61 mmHg. At that time, she had normal cardiopulmonary physical examination. 

A workup for PAH started which revealed tachycardia in electrocardiogram (ECG), and normal chest X-ray, spirometry and ventilation-perfusion scan of lungs. Laboratory investigations for vasculitis were negative. She had low level of thyroid stimulating hormone (TSH), normal total T4 level and increased T3 resin uptake (T3RU) at this time. These findings interpreted as thyroiditis due to IFN-β1a treatment discontinued 7 days before. Her thyroid function returned to normal levels about 10 days later and her palpitation disappeared; propranolol gradually discontinued. She received 20 mg of mitoxantrone and also advised to perform right-heart catheterization (RHC) but she refused. So the cardiologist decided to follow up her. 

In 2010, before the third dose of mitoxantrone and 6 months after stopping IFN-β1a injections, she had no cardiopulmonary symptom, and normal physical examination of cardiopulmonary system. The second echocardiography done by the same cardiologist showed an ejection fraction of 58% for left ventricle, mild tricuspid regurgitation and PAH (PA pressure of 43 mmHg). No specific treatment was done for her PAH. She received 5 doses of 20 mg mitoxantrone every three months. The last echocardiography performed in 2012 (2 years after IFN-β discontinuation) showed normal PAP (25 mmHg). She has still normal cardiopulmonary function and examination, although her Expanded Disability Status Scale (EDSS) has increased to 6.5.

Since the initial discovery of interferon in 1957 as an integral part of innate immunity, many different molecules in this family have been recognized. All three classes of IFNs have been developed as pharmaceuticals and they have clinical indications in many medical specialties.^1^

IFN-β1b was the first disease-modifying therapy for treatment of RRMS approved in 1993 by the United States Food and Drug Administration (USFDA). Subsequently, intramuscular (IM) IFN-β1a (1997) and IFN-β1b (2002) were approved.^1^ IFN treatment is accompanied by a variety of adverse events. Relatively, frequent side effects include flu-like symptoms, menstrual disorders, transient laboratory abnormalities, injection-site reactions and psychiatric disorders. A number of possible rare side effects were reported after commercial application of the drugs which were not reported in pivotal trials. These include development and exacerbation of other autoimmune diseases (thyroiditis, immune thrombocytopenia, and anemia), capillary leak syndrome, anaphylactic shock and polyneuropathy.^1^ There are also reports of livedoreticularis and Raynaud phenomenon, renal thrombotic microangiopathy and malignant arterial hypertension in patients with MS receiving IFN-β treatment.^2^

Since 1993, there are reports of PAH associated with IFN-α injections.^3^ The first report of PAH following IFN-β treatment was in 2009;^4^ after that, other cases of patients with MS were detected who developed this serious condition after IFN-β treatment,^3^^-^^7^ but this side effect has been remained rare among IFN-β users. In most of the IFN-α associated PAHs, there are also other risk factors of PAH; although some patients has not any other risk factors and in some, PAH has developed before IFN-α therapy, but progressed with this treatment to more severe disease.^3^

In the few reported cases of IFN-β-associated PAH, only one had a definite risk factor of PAH (atrial septal defect) which resolved after surgical repair.^3^ In other cases, PAH was severe and making combination treatment of was necessary, leading to death in 2 cases.^3^ In all previous cases, PAH was developed after more than 3 years of IFN-β injections. Our case is the first asymptomatic, incidentally detected PAH which was also self-limited after discontinuing the treatment. The interval time between starting IFN-β treatment and PAH detection was less than one year. 

Many experimental studies have investigated the effect of IFN on pulmonary vascular systems. Stimulation of thromboxane cascade, endothelin release, and “sensitizing” effect of tumor necrosis factor (TNF) on IFN responsiveness are all presumed mechanism according to the studies.^3^ But, the exact mechanism is to be determined. 

Although in many cases of IFN-α related PAH there are other risk factors for development of PAH, and IFN treatment seems to be a “trigger”, but as mentioned, there are some “pure” cases of PAH after IFN-β therapy, and IFN is now considered as a “possible” risk factor for PAH.^3^ As PAH is a serious condition, it seems reasonable to conduct well designed, clinical and experimental research to better understand the underlying mechanism of IFN-related PAH.

## Notes:

**How to cite this article:** Baghizadeh S, Sahraian MA, Ghahari M. Reversible pulmonary artery hypertension in association with interferon-beta therapy for multiple sclerosis. Iran J Neurol 2016; 15(1): 54-6.
